# Optical Polarization Sensor Based on a Metalens

**DOI:** 10.3390/s22207870

**Published:** 2022-10-17

**Authors:** Victor Kotlyar, Anton Nalimov, Alexey Kovalev, Sergey Stafeev

**Affiliations:** 1 IPSI RAS—Branch of the FSRC “Crystallography and Photonics” RAS, Molodogvardeyskaya 151, 443001 Samara, Russia; 2 Technical Cybernetics Department, Samara National Research University, Moskovskoye Shosse 34, 443086 Samara, Russia

**Keywords:** polarization sensor, sharp focusing, high-aperture metalens, topological charge

## Abstract

We investigated an optical microsensor of the polarization state of a laser light based on a metalens. In contrast to known polarization sensors based on metasurfaces that deflect different polarization types using various angles to the optical axis, the studied polarization sensor generated different patterns in the metalens focus to realize varied polarization states: left circular polarization generated a light ring in the focus, right circular polarization generated a circular focal spot, and linear polarization generated an elliptic spot with two sidelobes. Moreover, the tilt angle of the linear polarization matched the tilt angle of the elliptic focal spot. The simulation results were consistent with the theoretical predictions. A metalens with a diameter of several tens of microns was designed and fabricated in a thin amorphous silicon film with a thickness of 120 μm and a low aspect ratio, high numerical aperture, and short focal distance equal to a wavelength of 633 nm.

## 1. Introduction

Fabrication of compact high-performance optical sensors for controlling polarization of light is a complicated task. Traditional methods of polarization control such as linear polarizers, waveplates, and polarization modulators are typically not compact and require several measurements [1].

Among the compact and fast optical polarization sensors are polarization-imaging devices [2], optical [3] and plasmonic [4] on-chip polarimeters, and chiral beamsplitters based on gyroid photonic crystals [5]. However, the most effective and compact polarization sensors are those based on metasurfaces [6,7,8,9,10,11]. Metasurfaces are universal optical components because they allow the manipulation of all light properties (amplitude, phase, and polarization) simultaneously. All of the considered metasurfaces in [6,7,8,9,10,11] were based on a principle of spatial separation of different polarization states. In [6], a metasurface composed of parallelepiped nanopillars was fabricated in an amorphous silicon film with a thickness of 1680 nm. The pillars had a length of 285 nm, a width of 55 nm, and a height of 1550 nm. The wavelength of light was 974 nm. Orientation of the pillars controlled the phase delay. The metasurface acted as a diffraction grating (with a period of 3 μm): left circular polarization was directed into the –1st diffraction order, right circular polarization is directed into +1st order, and linear polarization was split into both +1st and −1st orders. The drawbacks of such a polarization sensor were a high aspect ratio of the nanopillars (1550:55), an absence of the focusing metalens, and the fact that registering the optical signal required photosensors separated in space. In [7], a dielectric gradient metasurface optical element was fabricated that operated like a blazed grating in a silicon layer that was 100 nm thick on a SiO_2_ substrate at a wavelength of 550 nm. Different states of polarization were directed by the meta-grating to different orders of diffraction. In [8], a similar gradient metasurface was fabricated as a blazed grating as in [7], but it was composed of gold nanorods that were 40 nm in height. The grating in [8] operated similarly to the grating in [7]. In [9], a hologram with a metasurface was fabricated that consisted of three layers: the upper layer was a set of silver nanorods, then a SiO_2_ spacer and a silver background layer were applied on the silicon substrate. The size of the meta-atom was 450 × 450 nm and the wavelength was 633 nm. When the meta-hologram was illuminated by a left circularly polarized radiation, the +1st order of the reflected light contained an image of a “bee”, whereas the −1st order contained an image of a “flower”. In [10], a metalens was created for multispectral chiral imaging that consisted of TiO_2_ nanofins on a glass substrate. The metalens operated in almost the entire visible wavelength range, but the length of the focus changed from 22 mm to 34 mm. In [11], a reflecting metasurface was fabricated that consisted of gold nanorods on a SiO_2_ substrate. The wavelength was 650 nm. This metasurface was intended for use in determining the tilt of the major axis of the polarization ellipse to the horizontal axis.

In this work, we investigated a metalens composed of sector-shaped binary subwavelength gratings in an amorphous silicon film. This metalens was free of the drawbacks listed above: the relief depth was only 120 nm, the metalens focused radiation at a distance of one wavelength (633 nm), and the photosensors were concentrated in the area of the focal spot. This polarization sensor worked in the following way: a light with left circular polarization was focused into a light ring (with zero intensity in the center), then a light with right circular polarization generated a circular focal spot, and a light with linear polarization generated an elliptic focal spot with two sidelobes.

## 2. Theoretical Background

Here, we consider a metasurface on which each point rotates a polarization vector of the incident laser light by an angle equal to the double polar (azimuthal) angle 2*φ* and adds a phase delay in the incident light that also is proportional to the polar angle. The transmittance of such a metalens is described by the following Jones matrix:

(1) $$\hat{T}(\varphi )=\exp\left(i\varphi \right)\left(\begin{array}{l} \cos2\varphi \;\;-\sin2\varphi \\ \sin2\varphi \;\;\;\;\;\cos2\varphi \; \end{array}\right)$$

If the metasurface (1) is illuminated by a left circularly polarized light, then the light that passes through such a surface also has left circular polarization:

(2) $$\left(\begin{array}{l} E_{xl}\\ E_{yl} \end{array}\right)=\hat{T}(\varphi )\left(\begin{array}{l} 1\\ -i \end{array}\right)=\exp\left(i\varphi \right)\left(\begin{array}{l} \cos2\varphi \;\;-\sin2\varphi \\ \sin2\varphi \;\;\;\;\;\cos2\varphi \; \end{array}\right)\left(\begin{array}{l} 1\\ -i \end{array}\right)=\exp(i3\varphi )\left(\begin{array}{l} 1\\ -i \end{array}\right)$$

where *E_x_* and *E_y_* are the horizontal and vertical components, respectively, of the transverse vector of the electric field strength. If a light field (2) is focused by a high-numerical-aperture ideal spherical lens, then in the focal plane, a light ring is generated. Indeed, as was shown in [12,13], if a light field has the following initial transverse amplitude:

(3) $$\left(\begin{array}{l} E_{x}\\ E_{y} \end{array}\right)=\frac{A(\theta )e^{im\varphi }}{\sqrt{2}}\left(\begin{array}{l} 1\\ i\sigma \end{array}\right)$$

where *σ* = 1 for right circular polarization, *σ* = −1 for left circular polarization, *σ* = 0 for linear polarization, and *σ* ≠ 0, ±1 for elliptical polarization; *A*(*θ*) is the angular spectrum of the initial field; and *θ* is the zenith angle (two angles *φ* and *θ* define a point on a sphere), then the intensity distribution in the sharp focus is given as:

(4) $$\begin{array}{l} I_{m,\pm }=\left(\frac{1+\sigma^{2}}{8}\right)I_{0,m}^{2}+\frac{\gamma_{+}^{2}}{4}\left(2I_{1,m+1}^{2}+I_{2,m+2}^{2}\right)\\ +\frac{\gamma_{-}^{2}}{4}\left(2I_{1,m-1}^{2}+I_{2,m-2}^{2}\right)+\cos2\varphi \left(\frac{1-\sigma^{2}}{8}\right)\\ \times \left(I_{0,m}I_{2,m+2}+I_{0,m}I_{2,m-2}-2I_{1,m-1}I_{1,m+1}\right). \end{array}$$

where $\gamma_{\pm }=\left(1\pm \sigma \right)/2$ and at *σ*= 0 these values are equal ($\gamma_{+}=\gamma_{-}=1/\sqrt{2}$) and the functions *I_ν,μ_* depend solely on the radial coordinate *r* in the focal plane (*z* = 0) and at any *z* are equal to:

(5) $$I_{\nu ,\mu }=\left(\frac{\pi f}{\lambda }\right)\int_{0}^{\theta_{\max}}\sin^{\nu +1}(\frac{\theta }{2})\cos^{3-\nu }(\frac{\theta }{2})\cos^{1/2}(\theta )A(\theta )e^{ikz\cos\theta }J_{\mu }(x)d\theta ,$$

where *λ* is the wavelength of light; *f* is the focal length of an aplanatic system; *x = kr*sin*θ*; *J_μ_*(*x*) is a Bessel function of the first kind; and *NA =* sin *θ*_max_ is the numerical aperture. The original amplitude function *A*(*θ*) (assumed here to be real) may be constant (for a plane wave) or described by a Gaussian beam. For the initial field (2), it should be assumed in Equation (4) that *m* = 3, *γ*_+_ = 0, *γ_−_* = 1, and *σ*^2^ = 1. Then, we get an expression for the focal intensity distribution of the initial field (2):

(6) $$I_{3,-}=\frac{1}{4}\left(I_{0,3}^{2}+2I_{1,2}^{2}+I_{2,1}^{2}\right).$$

According to Equation (6), in the center of the focus (on the optical axis) at *r* = 0, the intensity is equal to zero because the second index of the functions *I_ν,μ_* is equal to the order of the Bessel function, which equals zero at *r* = 0 if the order is μ ≠ 0. Equation (6) also indicates that the focal intensity pattern has a circular symmetry because the intensity depends only on the radial coordinate *r*. Thus, when a light field with left circular polarization with the Jones vector (1, −*i*)*^T^*, (*T* means the transposing) passes through a metasurface with the transmittance (1), it is converted into an third-order optical vortex with left circular polarization (2). In the sharp focus, such a light beam generates a light ring with zero intensity in the center (6).

If the metasurface (1) is now illuminated by a light with right circular polarization, then an optical vortex of the minus first order is generated at the output with right circular polarization:

(7) $$\left(\begin{array}{l} E_{xr}\\ E_{yr} \end{array}\right)=\hat{T}(\varphi )\left(\begin{array}{l} 1\\ i \end{array}\right)=\exp\left(i\varphi \right)\left(\begin{array}{l} \cos2\varphi \;\;-\sin2\varphi \\ \sin2\varphi \;\;\;\;\;\cos2\varphi \; \end{array}\right)\left(\begin{array}{l} 1\\ i \end{array}\right)=\exp(-i\varphi )\left(\begin{array}{l} 1\\ i \end{array}\right)$$

Equation (4) allows the finding of the intensity distribution in the sharp focus for the initial field (7). To do this, we supposed in Equation (4) that *m* = −1, *γ*_+_ = 1, *γ_−_* = 0, and *σ*^2^ = 1. Then, the focal intensity for the initial field (7) is given as:

(8) $$I_{-1,+}=\frac{1}{4}\left(I_{0,1}^{2}+2I_{1,0}^{2}+I_{2,1}^{2}\right).$$

The second term in Equation (8) contains a function *I*_1,0_ with the second index equal to zero. This means that the integral in Equation (5) contains the zero-order Bessel function, which is not zero at *r* = 0. Thus, there is a maximum in the center of the focal intensity distribution and the focus has a shape of a circular light spot.

If the metasurface (1) is now illuminated by a light with linear polarization along the axis *y*, then there is an optical vortex of the +1st order at the output with second-order azimuthal polarization:

(9) $$\left(\begin{array}{l} E_{x}\\ E_{y} \end{array}\right)=\hat{T}(\varphi )\left(\begin{array}{l} 0\\ 1 \end{array}\right)=\exp\left(i\varphi \right)\left(\begin{array}{l} \cos2\varphi \;\;-\sin2\varphi \\ \sin2\varphi \;\;\;\;\;\cos2\varphi \; \end{array}\right)\left(\begin{array}{l} 0\\ 1 \end{array}\right)=\exp(i\varphi )\left(\begin{array}{l} -\sin2\varphi \\ \cos2\varphi \end{array}\right)$$

For the initial field (9), Equation (4) does not allow obtaining the focal intensity distribution. In [12], a general expression was derived for the focal intensity for an initial optical vortex with the topological charge of *m* and with *n*th-order azimuthal polarization that had the Jones vector:

(10) $$E=A(\theta )e^{im\varphi }\left(\begin{array}{l} -\sin n\varphi \\ \cos n\varphi \end{array}\right)$$

In the sharp focus of an aplanatic system, the intensity distribution for the initial field (10) reads as:

(11) $$\begin{array}{l} I_{}=\frac{1}{2}\left(I_{0,m+n}^{2}+I_{0,m-n}^{2}+I_{2,m+n-2}^{2}+I_{2,m-n+2}^{2}\right)+I_{1,m+n-1}^{2}+I_{1,m-n+1}^{2}+\\ +{\left(-1\right)}^{n+1}\left(I_{0,m+n}I_{2,m-n+2}+I_{0,m-n}I_{2,m+n-2}-2I_{1,m+n-1}I_{1,m-n+1}\right)\cos2\left(n-1\right)\varphi . \end{array}$$

Based on Equation (11), we get the focal intensity distribution for the initial field (9), assuming in Equation (11) that *m* = 1, *n* = 2:

(12) $$\begin{array}{l} I=\frac{1}{2}\left(I_{0,3}^{2}+I_{0,1}^{2}+2I_{2,1}^{2}\right)+I_{1,2}^{2}+I_{1,0}^{2}+\\ -\left(I_{0,3}I_{2,1}-I_{0,1}I_{2,1}-2I_{1,2}I_{1,0}\right)\cos2\varphi . \end{array}$$

According to Equation (12), the intensity in the center of the focus on the optical axis (*r* = 0) is not zero because Equation (12) contains the term *I*_1,0_. This term has the zero second index and the integral in Equation (5) contains the zero-order Bessel function, which equals 1 at zero argument. However, the focal spot is not circularly symmetric, since Equation (12) is dependent on the polar angle as cos2*φ*. Thus, in addition to the central maximum, the intensity distribution (12) contains two additional maxima (two sidelobes) residing on the vertical axis at *φ* = *π*/2 and *φ* = *3π*/2. Since cos2*φ* = −1 at these angles, instead of Equation (12) we get the following expression:

(13) $$I=\frac{1}{2}{\left(I_{0,3}+I_{2,1}\right)}^{2}+\frac{1}{2}{\left(I_{0,1}-I_{2,1}\right)}^{2}+{\left(I_{1,2}-I_{1,0}\right)}^{2}.$$

We note that earlier in [12,13] we derived general expressions for a vortex field with a topological charge *m* and with circular polarization. As was demonstrated in [13], when a circularly polarized conventional Gaussian beam is sharply focused, spin–orbit interaction in the focus leads to rotation of the transverse energy flow or, in other words, a longitudinal component of the orbital angular momentum vector appears. In this work, based on [12,13], new expressions were obtained for describing three light fields in the sharp focus: an optical vortex with the topological charge *m* = 3 and with right circular polarization (Equation (6)), an optical vortex with the topological charge *m* = −1 and with left circular polarization (Equation (8)), and an optical vortex with the topological charge *m* = 1 and with second-order azimuthal polarization (Equations (12) and (13)).

Thus, we have shown theoretically that using a metasurface and a high-numerical-aperture focusing lens allows easy detection of different homogeneous polarization states of a laser beam. If a surface (1) is illuminated by a light field with left circular polarization, then the focus contains a light ring with zero intensity in the center. If a surface (1) is illuminated by a light field with right circular polarization, then the focus contains a circular light spot with the intensity maximum in the center. Finally, if a surface (1) is illuminated by a linearly polarized light, then there is an elliptic spot in the focus accompanied with two sidelobes. In addition, the segment between the centers of these two sidelobes indicates the direction of the linear polarization vector of the initial light field. The numerical simulation, described in the next section, confirmed the theoretical outcomes.

## 3. Numerical Simulation

**Figure 1:**
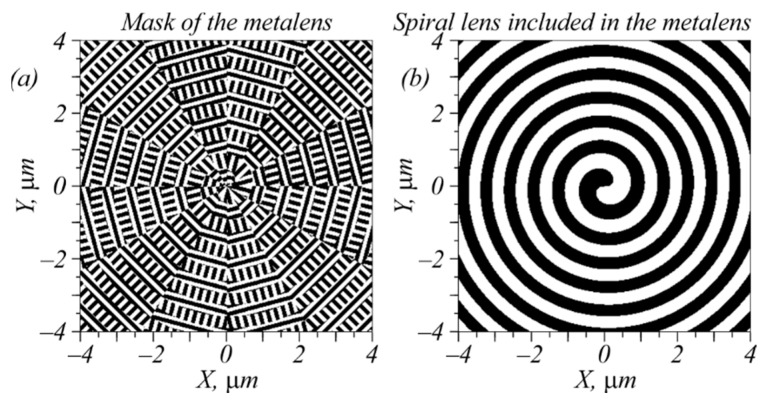
A view of the metalens under consideration, which had a size of 8 × 8 μm (**a**), and a spiral zone plate included in the metalens (**b**).

In this section, we designed a metalens composed of sector-shaped subwavelength gratings with a transmittance function described by the matrix in Equation (1) and by a spiral binary zone plate with a high numerical aperture. The first-order spiral zone plate (Figure 1b) generated an optical vortex with the topological charge +1 and focused a light field passed through the plate. The zone plate should have a high numerical aperture (close to unit) so that, due to the spin–orbit interaction, three amplitudes of the initial light fields (2), (7), and (9) will lead to different focal spots in the sharp focus. Figure 1a illustrates the considered metalens designed with the following parameters: wavelength of light λ = 633 nm, focal length of the metalens *f* = λ, metalens material is amorphous silicon (with a refractive index of *n* = 4.33 + 0.486*i*), and relief height = 120 nm. The metalens was composed of sector-shaped binary subwavelength diffraction gratings (with a period of 220 nm) with grooves and ridges that both had a width of 110 nm. The incident light field was bounded by a circular aperture with a radius of 4 μm. Such a small size of the metalens was chosen to simplify the computations. A detailed description of the design of such sector metalenses can be found in [14]. Figure 1b shows a template of the binary spiral zone plate included in the metalens shown in Figure 1a.

**Figure 2:**
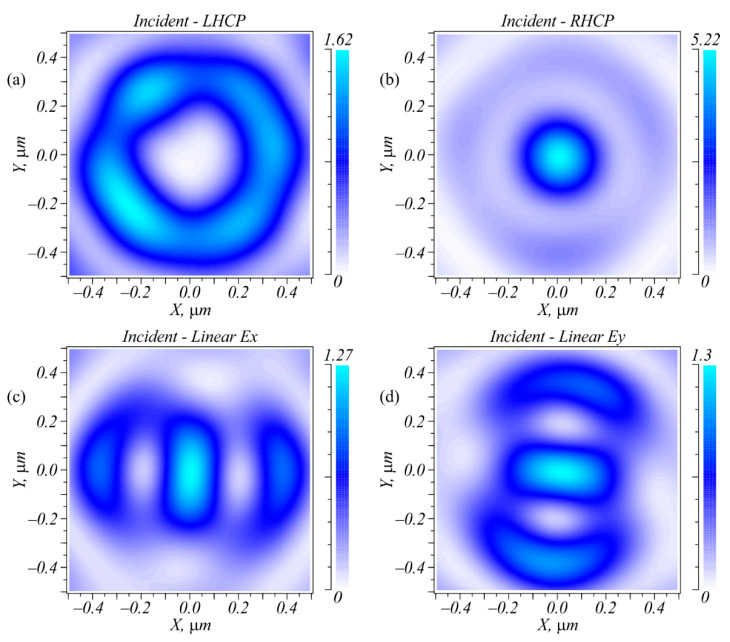
Intensity distributions in the metalens focus (Figure 1) for four different polarizations of the incident field: LHCP (**a**); RHCP (**b**); linear *Ex* (**c**); linear *Ey* (**d**).

**Figure 3:**
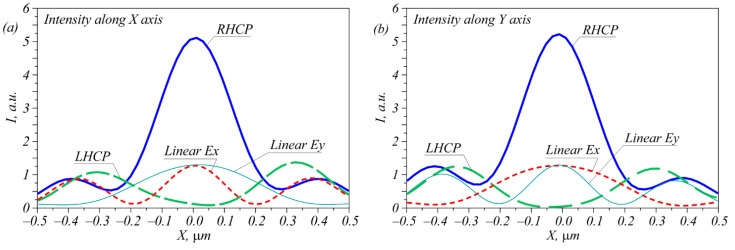
Intensity profiles for all four polarizations along the X (**a**) and Y (**b**) axes.

The simulation was carried out using the difference solution of the Maxwell equations via the finite difference time domain method (FDTD method) implemented in the Fullwave program (Rsoft). The simulation grid along all three coordinates in space was equal to λ/30. Figure 2 depicts the intensity distributions of laser beams with four polarization types; i.e., left circular (LHCP), right circular (RHCP), and linear polarization with the electric strength vector being directed along the X and Y axes after passing through the metalens (Figure 1). Figure 3 shows the intensity cross-sections along the X and Y axes.

As can be seen in Figure 2 and Figure 3, all four polarizations generated focal spots that differed in both the shape and the amplitude. Right circular polarization generated a circular focal spot (Figure 2b) with a diameter at the half-maximum intensity equal to FWHM = 0.427λ. Left circular polarization generated in the metalens focused (Figure 1) a light ring with a radius of 0.31 μm and a ring thickness of 0.4λ. When focusing a linearly polarized light, an elliptic focal spot was generated with the widths at half-maximum intensity equal to FWHM = 0.32λ along the small axis of the ellipse and FWHM = 0.68λ along the large axis of the ellipse. Note that the light ring shown in Figure 2a was not smooth because the transmittance function of the inclined diffraction gratings did not have a constant value.

**Figure 4:**
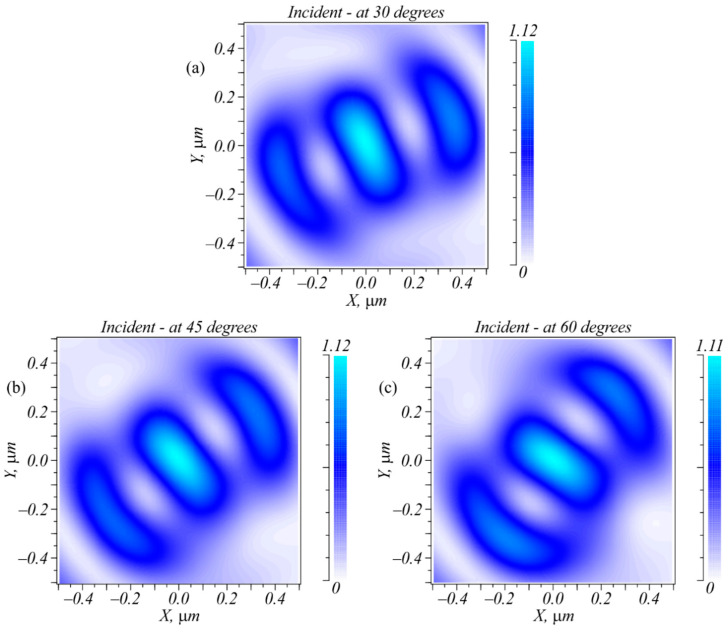
Intensity distribution at the focal plane for the incident linear polarization rotated at angle φ: 30° (**a**); 45° (**b**); 60° (**c**).

Putting one or two intensity photodetectors in the focal plane allowed the detection of beam polarization. For instance, measuring only the focal spot intensity in the center allowed unambiguous detection of three polarization types: LHCP, RHCP, and linear *E_x_* or *E_y_*. To detect the direction of linear polarization, one additional sensor was needed that was located on the X- or Y-axis at a distance of 0.38 μm from the center. According to Figure 2, when the incident field was linearly polarized, the focal plane contained an elliptic focal spot with sidelobes. If the electric strength vector of the incident field was rotated, this spot was also rotated around its axis so that the sidelobes resided along the oscillation axis of the electric strength vector of the initial field. This is demonstrated in Figure 4. Thus, an arbitrary direction of linear polarization could be detected if there were enough intensity sensors located on the radius of 0.38 μm.

**Figure 5:**
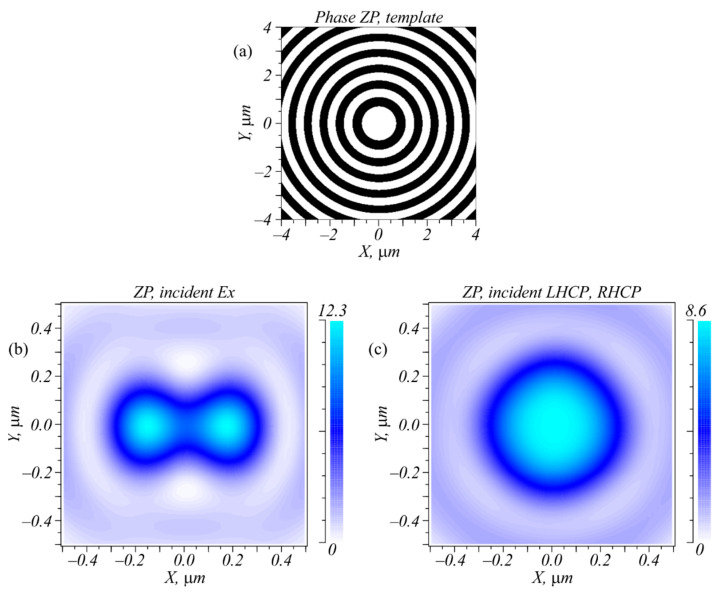
A template of the phase binary zone plate (**a**) and intensity distributions in the focal plane (at a distance *f* = λ = 633 nm) for a light field with linear polarization along the X-axis (**b**) and with circular (left and right) polarization (**c**).

To complete the picture, below we outline our study of focusing of light with different polarization by using a binary zone plate with the same numerical aperture as that of the metalens. The numerical apertures of both lenses were close to unity. Figure 5a shows the binary template of the zone plate (black rings—phase is π; white rings—phase is zero). Figure 5b shows the focal spot with a dumbbell shape for a light with linear polarization along the X-axis and Figure 5c shows the circular focal spot for left and right circular polarization. Figure 5 indicates that it was impossible to distinguish left and right polarization in the focus of an ordinary zone plate.

A comparison of Figure 2b and Figure 5b revealed an interesting effect: the diameter of the focus of a circularly polarized light was equal to FWHM = 0.826λ (Figure 5b) and was nearly two times larger than the diameter of the focus for an optical vortex with a topological charge of −1 and with right circular polarization (FWHM = 0.427λ) (Figure 2b) at the same numerical aperture of 1. This occurred because the focus in Figure 2b was generated mainly by the longitudinal component *E_z_* of the electric field, whereas Figure 5b shows that the longitudinal component had the shape of rings and widened the focal spot. The *E_x_* and *E_y_* projections shown in Figure 5b had the shape of spot.

## 4. Experiment

As an example, a simplified metalens was fabricated using e-beam lithography and ion-beam etching in a thin amorphous silicon film. Its transmittance was different from the transmittance of the metalens (1) only in the absence of the vortex factor:

(14) $${\hat{T}}_{1}(\varphi )=\left(\begin{array}{l} \cos2\varphi \;\;-\sin2\varphi \\ \sin2\varphi \;\;\;\;\;\cos2\varphi \; \end{array}\right)$$

The metalens (14) acted similarly to the metalens (1) if it was illuminated by an optical vortex with a topological charge of +1 and a corresponding polarization. This meant that the focal pattern of the metalens (14) contained a ring, a circular spot, or an elliptic spot if the metalens (14) was illuminated by a light field with the Jones vectors respectively equal to:

(15) $$\exp(i\varphi )\left(\begin{array}{l} 1\\ -i \end{array}\right),\;\;\exp(i\varphi )\left(\begin{array}{l} 1\\ i \end{array}\right),\;\;\exp(i\varphi )\left(\begin{array}{l} 1\\ 0 \end{array}\right).$$

We intentionally fabricated metalens (14) instead of metalens (1) because it was more universal. Metalens (14) could be illuminated by optical vortices with the topological charges different both in the magnitude and in the sign, as well as by cylindrical vector beams with radial and azimuthal polarization.

**Figure 6:**
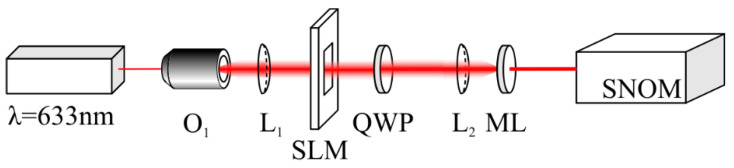
Experimental setup. Laser—Cobolt 06 MLD (λ = 633 nm, 80 mW); O_1_—20× objective lens; SLM—spatial light modulator (Holoeye LC 2012, 1024 × 768 px); L_1_, L_2_—lenses (f_1_ = 150 mm and f_2_ = 10 mm), QWP—quarter-wave plate; SNOM—scanning near-field optical microscope (Ntegra Spectra).

Figure 6 shows the experimental setup. A linearly polarized light beam from a solid-state laser with a wavelength of 633 nm and with a power of 80 mW was extended and collimated by a microscope objective (O_1_) and by a spherical lens (L_1_). After passing through the SLM, an optical vortex of the +1 order was generated. A quarter-wave plate (QWP) transformed the linear polarization into circular and the spherical lens (L_2_) focused the laser light onto the metalens (ML). In the metalens focus, there was a cantilever of a scanning near-field optical microscope (SNOM). The cantilever was a hollow metallic pyramid with a hole in the vertex with a 100 nm diameter. The light that passed through this subwavelength hole of the cantilever was registered by a CCD camera. The transverse scanning step by the cantilever was nearly 30 nm. Thus, the intensity pattern in the metalens focus was measured with a space resolution of nearly 30 nm.

**Figure 7:**
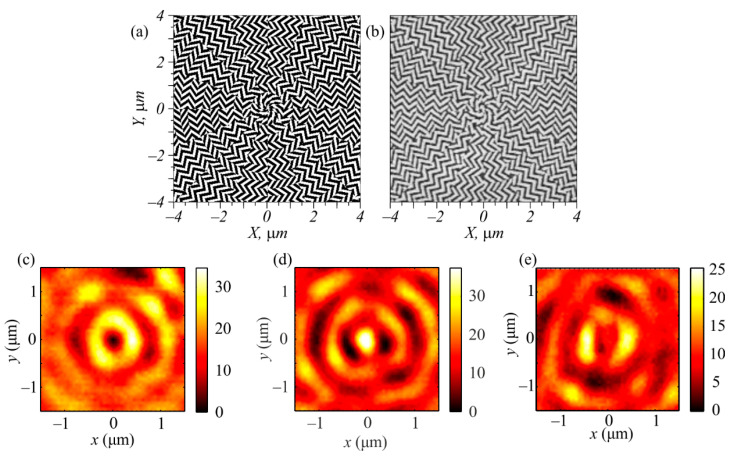
Binary photo template of metalens (14) (**a**); central part of the metalens fabricated in an amorphous silicon film (**b**); intensity distribution in the focus of the metalens when it was illuminated by an optical vortex with a topological charge of +1 and with different polarizations: left circular (**c**), right circular (**d**), and linear (along the horizontal axis) (**e**).

Figure 7a illustrates a binary template of a metalens that had a transmittance of the metasurface (14) combined with the zone plate (Figure 5a). The other parameters were the same as for the metalens shown in Figure 1. Figure 7b shows a scanning electron microscope (SEM) image of the central part of the metalens that was fabricated according to the template shown in Figure 7a. The metalens shown in Figure 7b, which had a diameter of 30 μm, was fabricated in an amorphous silicon film with a thickness of 120 nm and deposited onto a Pyrex substrate (with a refractive index of 1.5). The film was coated with a 150 nm ZEP resist layer that was baked at 180 °C. To dissipate the charge, a 10 nm aluminum coating was applied onto the sample surface. The binary pattern was transferred onto the resist with a 30 kV electron beam. The sample was then developed in ZED-N50. Since the amorphous silicon absorbed visible light significantly, the effectivity of this metalens was not very high (10−15%), but it had a low aspect ratio (120:110) in contrast to other metalenses. For example, in [6], the ratio between the relief depth and the transverse section of the nanopillar was too high (1550:55). This made the metalens fabrication rather difficult.

Figure 7c–e depict the intensity distributions registered in the focus of the metalens (Figure 7b) at a distance of *f* = λ = 633 nm by a scanning near-field optical microscope (SNOM) made by NT-MDT when the metalens was illuminated by an optical vortex with a topological charge of +1 and with left circular, right circular, or linear polarization, respectively. The optical vortex was generated by the SLM (Holoeye LC 2012). The focal patterns shown in Figure 7c–e were qualitatively consistent with the patterns shown in Figure 2a–c. The focal spots shown in Figure 7c–e had characteristic sizes greater than those shown in Figure 2a–c. In the simulation, the diameter of the focus was equal to 0.427λ (Figure 2b), the ring diameter was 0.62 μm (Figure 2a), and the distance between the sidelobes was 0.8 μm (Figure 2c). In the experiment, these values were greater by 20%: 0.62λ (Figure 7d), 0.8 μm (Figure 7c), and 1 μm (Figure 7e), respectively. The greater sizes of the focal spots in the experiment compared to those in the simulation were explainable because in the experiment, the metalens was placed into the diverging area of the Gaussian optical vortex rather than into the waist.

## 5. Conclusions

We theoretically, numerically, and experimentally investigated a polarization microsensor based on a high-numerical-aperture metalens in a thin silicon film. Other known similar metalens-based polarization sensors [6,7,8] did not include the focusing lens and deflected the light with different polarizations using different angles. This led to increased sensor sizes and required the use of a photosensor matrix (CCD camera). The polarization sensor considered here had a size of just tens of microns, focused a light at a distance of one wavelength, and was based on a different principle. Instead of deflecting the light with different polarizations using different angles, this sensor generated different diffraction patterns in the focus on the optical axis (circular focal spot, light ring, or elliptic focal spot with sidelobes). This allowed us to replace the photosensor matrix with just one or two photosensors with a sensing area of several hundred nanometers. The polarization sensor considered here can find applications for controlling the polarization states in biology [15], medicine [16,17], and microscopy [18,19].

## Data Availability

Not applicable.
